# *NORAD*-Regulated Signaling Pathways in Breast Cancer Progression

**DOI:** 10.3390/cancers16030636

**Published:** 2024-02-01

**Authors:** Ana Maria Capela, Carlota Tavares-Marcos, Hugo F. Estima-Arede, Sandrina Nóbrega-Pereira, Bruno Bernardes de Jesus

**Affiliations:** Department of Medical Sciences, Institute of Biomedicine—iBiMED, University of Aveiro, 3810-193 Aveiro, Portugal; anammcapela@ua.pt (A.M.C.); carlotamarcos3@ua.pt (C.T.-M.); hugoarede@ua.pt (H.F.E.-A.)

**Keywords:** lncRNAs, *NORAD*, pumilio, breast cancer, chemotherapy resistance

## Abstract

**Simple Summary:**

Breast cancer (BC) is a heterogeneous disease classified into different subtypes presenting several treatment challenges, especially in more advanced cases arising from triple negative breast cancer. *NORAD* is a long non-coding RNA (lncRNA) activated by DNA damage, with an impacting role in the repair process of DNA insults. This lncRNA is differentially expressed in BC subtypes, participating in cancer initiation and progression, by interacting with an extended range of signaling partners. Here, we review the network of *NORAD* molecular interactions with relevance, as well as *NORAD*’s potential as a prognostic, predictive and target for BC treatment.

**Abstract:**

Long non-coding RNA activated by DNA damage (*NORAD*) has recently been associated with pathologic mechanisms underlying cancer progression. Due to *NORAD*’s extended range of interacting partners, there has been contradictory data on its oncogenic or tumor suppressor roles in BC. This review will summarize the function of *NORAD* in different BC subtypes and how *NORAD* impacts crucial signaling pathways in this pathology. Through the preferential binding to pumilio (PUM) proteins PUM1 and PUM2, *NORAD* has been shown to be involved in the control of cell cycle, angiogenesis, mitosis, DNA replication and transcription and protein translation. More recently, *NORAD* has been associated with PUM-independent roles, accomplished by interacting with other ncRNAs, mRNAs and proteins. The intricate network of *NORAD*-mediated signaling pathways may provide insights into the potential design of novel unexplored strategies to overcome chemotherapy resistance in BC treatment.

## 1. Introduction

Long non-coding RNAs (lncRNAs) are a class of non-protein coding RNAs longer than 200 nucleotides in length and with limited or no detectable open reading frame (ORF) [1,2]. Despite being considered for decades as “junk RNA” [3], they were found to control transcriptional gene expression, translational and post-translational events [4]. The mechanism behind lncRNA function spans from the modulation of chromatin conformation, interaction with transcription factors, binding to RNA-binding proteins (RBPs) and messenger RNAs (mRNAs) or even acting as competitive endogenous RNA (ceRNA) by sponging microRNAs (miRNAs) [5,6]. Hence, they participate in several biological processes, namely in alternative splicing, epigenetic regulation, RNA decay and protein translation [7]. Most lncRNAs are transcribed by RNA polymerase II, capped at their 5′ end and then spliced and polyadenylated at their 3′ end, similar to mRNA [8,9]. They are generally expressed at low levels, tissue-specific and poorly conserved, and have been shown to influence physiological and pathological conditions, in particular neurological disorders, aging and cancer [6].

Long non-coding RNA activated by DNA damage (*NORAD*) was first discovered by Lee et al., when exploring the role of lncRNAs in genomic stability regulation [10]. *NORAD* is a 5.3 kb, highly conserved lncRNA, localized on chromosome 20 (20q11.23) and located in the cytoplasm, accumulating in the nucleus during replication, stress and DNA damage [1,11]. Various studies link *NORAD* to genomic stability as, in its absence, cells acquire a chromosomal instability (CIN) phenotype and aneuploidy [12]. *NORAD*’s main mechanism of action is binding to pumilio (PUM) proteins. These proteins repress several mRNA transcripts involved in germline homeostasis, cell cycle control and neuronal activity and function that are required for adequate mitosis, DNA repair and replication [1,10]. Mechanistically, *NORAD* sequesters PUM proteins, preventing their mRNA targets’ inhibition and leading to chromosomal stability maintenance [10,13]. Apart from PUM proteins, recent advances have highlighted other *NORAD*-interacting partners [1,11], such as proteins involved in different signaling pathways, particularly STAT, TGF-β, Akt/mTOR and PI3K/Akt pathway and miRNAs [14].

Due to *NORAD*’s extensive network of binding partners, it has been associated with different pathological conditions including cardiovascular, cerebrovascular and degenerative diseases [15,16,17], but mostly with cancer [1]. Studies suggest that *NORAD* is dysregulated in numerous cancers, including breast, renal, gastric, bladder, pancreatic, ovarian, cervical, prostate, lung and endometrial cancer [18]. Most of the studies describe *NORAD* as being overexpressed, leading to cancer cell proliferation, invasion and metastatic behavior [18]. In addition, this lncRNA has been associated with resistance to chemotherapeutic agents used in clinical practice, such as 5-fluorouracil in colorectal cancer, gemcitabine in bladder cancer and doxorubicin in neuroblastoma [19,20,21].

Breast cancer (BC) is the most diagnosed cancer worldwide [22] and represents one of the malignancies in which *NORAD* expression is altered. BC can be classified into luminal A and B (LumA and LumB, respectively), human epidermal growth factor receptor 2 (HER2)-enriched and triple negative breast cancer (TNBC), according to the expression (or absence) of estrogen, progesterone and HER2 receptors and the level of Ki67 [23]. TNBC can be further stratified into more specific and intrinsic subtypes, such as the basal-like (BL) subtype [24]. BC treatment is decided according to the subtype, and the most common treatments are surgery, radiation, chemotherapy, hormonal therapy (for tumors that express estrogen and progesterone receptors), targeted therapy (especially directed to HER2 in HER2-enriched BC) and immunotherapy [25,26,27]. TNBC, however, has less targeted treatment options and is less responsive to chemotherapy [25,27], with a high number of patients presenting recurrence and metastasis [28]. In the last decades, more personalized and targeted molecular treatments have been developed, such as inhibitors of poly(ADP-ribose) polymerase (PARP), cyclin-dependent kinases (CDK) 4 and 6 and phosphatidylinositol 3-kinase (PI3K)/protein kinase B (PKB)/mammalian target of rapamycin (mTOR) pathway [29].

The role of *NORAD* in BC progression remains controversial [30]. *NORAD* is predominantly associated with oncogenic functions, displaying high expression in BC tumors, human cell lines and in the peripheral blood of BC patients [31]. This lncRNA is associated with increased cell proliferation, invasion and migration, tumor growth and size, worst clinical stage, histological grade and lymph node metastasis (LNM), leading to poor prognosis and reduced disease-free survival (DFS) in BC patients [30,31,32,33,34]. It is also correlated positively with metastasis and stemness and negatively associated with DNA repair and inflammation [31]. Additionally, *NORAD*-silenced cells present reduced invasion, migration, cell viability and colony formation [34], and xenograft BC mouse models established with *NORAD*-silenced cells present development of smaller tumors [35]. *NORAD* is also significantly upregulated in BC-derived exosomes, associated with increased m6A methylation [36,37]. In other studies, *NORAD* is considered a tumor suppressor as its expression levels are lower in BC tissues and cancer cells compared to normal conditions, leading to increased cell proliferation, migration and invasion, LNM development and poor prognosis [38,39]. In this context, *NORAD* overexpression (OE) in human BC cell lines leads to reduced migration and invasion, while *NORAD* silencing has the opposite effect [40]. *NORAD* is described, on one hand, to be more expressed in the LumA subtype as compared to BL [41,42], with the lowest levels in BL [39], and, related to luminal subtypes [31], and, on the other hand, to be more expressed in TNBC compared to LumA [43]. The studies agree, however, that *NORAD* is differentially expressed in distinct BC subtypes and is related to patient survival in the BL subtype [42,43].

**Figure 1:**
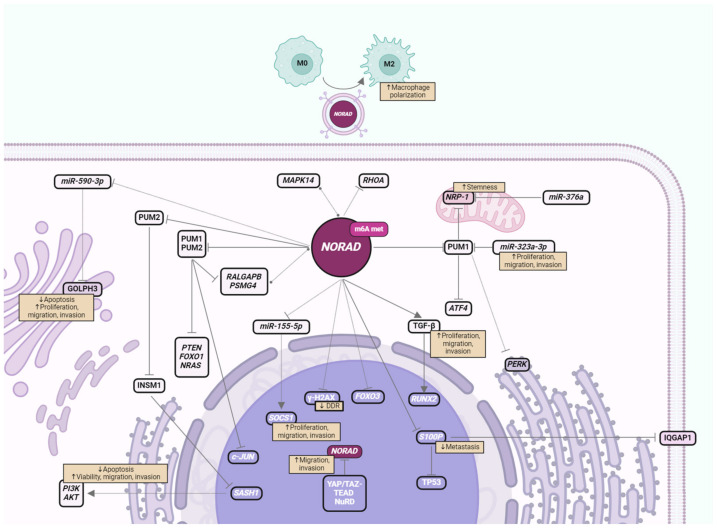
Schematic of *NORAD* molecular interacting partners in BC, emphasizing cellular localization, and impact on cancer progression. Solid lines indicate direct binding, dotted lines indicate indirect, undescribed or untested experimentally binding mechanism in BC, where circular ends refer to co-expression, cut ends to repression and pointed ends to promotion of expression. The impact on BC progression is also described, specifically how these interactions are reflected in cellular viability, proliferation, migration, invasion, apoptosis, metastatic capacity and stemness. In the nucleus, YAP/TAZ-TEAD and NuRD repress *NORAD* transcription, increasing migration and invasion of BC cells. *NORAD* also affects histone γH2AX expression and the consequent DNA damage response (DDR). In the cytoplasm, *NORAD* has increased expression and m6A methylation and is consequently secreted in exosomes to influence other cell types such as macrophage polarization into M2 protumoral phenotype. *NORAD* sequesters PUM1 and PUM2, leading to repression of *c-JUN* transcription and decreased levels of *PTEN*, *FOXO1*, *NRAS*, *RALGAPB* and *PSMG4* transcripts in the cytoplasm, where the latter two co-express with *NORAD*. *MiR-323a-3p* also binds PUM1 along with *NORAD*, resulting in increased *NRP-1* expression in the mitochondria, which is in turn repressed by *miR-376a*. eIF2 downstream effectors PERK on the rough endoplasmic reticulum and ATF4 are consequently induced. PUM2, sequestered by *NORAD*, also represses INSM1, which represses *SASH1* transcription, lowering *PI3K* and *AKT* levels. Independently of PUM, in the cytoplasm, *NORAD* represses *RHOA, miR-155-5p* (inducing nuclear *SOCS1* expression) and *miR-590-3P* (decreasing Golgi apparatus GOLPH3), induces TGF-β (increasing nuclear RUNX2) and co-expresses with *MAPK14*. *NORAD* also represses *S100P* transcription, decreasing S100P binding to TP53 and IQGAP1 proteins, and the amount of IQGAP1 in the membrane. Subcellular localization was based on UniProt data [44]. Created with BioRender.com.

There are several signaling pathways with relevance for BC progression that are affected by *NORAD* expression [14]. Understanding the impact and context-dependent interactions of *NORAD* in crucial signaling pathways may highlight *NORAD* as a relevant therapeutic option to treat BC and overcome therapy resistance. In this review, we will summarize *NORAD* interactions and their relevance in BC progression and treatment. This includes *NORAD* interactions that are well established in BC, both involving PUM proteins and ncRNA sponging, and also other interactions shown to be affected by *NORAD* where the exact mechanism is not yet well understood (Figure 1 and Table 1).

## 2. Impact of *NORAD* in BC Signaling Pathways

### 2.1. PUM Proteins and Target Genes

PUM proteins are RBPs from the highly conserved Puf family. In mammals, the two canonical cytoplasmic PUM proteins are pumilio RNA binding family member 1 and 2 (PUM1 and PUM2, respectively). PUM proteins bind specifically and with great affinity to the conserved motifs of Pumilio Recognition/Response Element (PRE) found in the 3′ Untranslated Region (UTR) of their target genes, and they post-transcriptionally regulate mRNA degradation and repress protein translation [54,55]. In some cases, PUMs can act in translation to prevent their target’s ubiquitination and increase protein stability [56]. Some PUM target genes, including *PARP1*, minichromosome maintenance complex component 4 (*MCM4*), the structural maintenance of chromosomes 1A (*SMC1A*) and centromere protein J (*CENPJ*), regulate important biological functions, such as DNA repair and replication, cell cycle and mitosis. *NORAD* was discovered and first described in the human colorectal cancer cell line HCT116 where in silico assays revealed repetitive sequences containing PREs, allowing for PUM1 and PUM2 binding [10]. After DNA damage induction, *NORAD* co-localizes with PUM in *NORAD*–PUM (NP) bodies in the cytoplasm where *NORAD* negatively regulates cytoplasmic PUM proteins in phase-separated condensates as ribonucleoprotein (RNP) granules. *NORAD*’s high expression and the presence of multiple PREs allows for complete and competitive PUM recruitment and the subsequent maintenance of genome stability [57]. PUM expression and its impact on BC is also being debated. Some studies report PUM1 to be one of the most differently expressed and methylated genes in BC [58] and PUM2 to have higher expression in tumors as in TNBC, where it negatively correlates with BC patient overall survival (OS) and relapse-free survival (RFS) [46]. Other studies report lower PUM2 expression in LumA and TNBC tumors compared to normal tissues and that PUM2 silencing increases cell viability, migration and invasion in cancer cells lines, while its OE produces the opposite effect [48]. Slight variations in the expression or availability of PUM proteins are sufficient to lead to CIN. In this context, the absence of *NORAD* leads to the release and hyperactivation of PUM proteins and the appearance of deleterious effects, such as accelerating premature aging in mice [12]. *NORAD* can sequester a significant fraction of PUM proteins, negatively regulating their capacity to repress target mRNAs [10]. In this line of thought, several PUM targets and their implications in BC progression will be further described below (summarized in Figure 1 and Table 1).

Ral GTPase activating protein non-catalytic subunit beta (RALGAPB) participates in the regulation of mitosis, and its dysregulation is associated with genomic instability [59]. In some cancers, such as pancreatic ductal adenocarcinoma (PDAC) and oral squamous cell carcinoma (OSCC), *RALGAPB* depletion has been reported to promote invasion, migration, tumor growth and metastasis by increasing transforming growth factor beta 1 (TGFB1) signaling and decreasing c-Jun N-terminal kinase activity [60,61] and mTORC1-dependent pancreatic tumor cell invasion [62,63]. Based on The Cancer Genome Atlas (TCGA) RNA-seq data on BC tissues and clinical data from the cBioPortal platform, PUM-binding lncRNAs were selected and evaluated in each BC subtype. Interestingly, *RALGAPB* was revealed to co-express with *NORAD* in all analyzed BC subtypes. The high expression of both *NORAD* and *RALGAPB* was associated with worse prognosis and poorer OS in LumA subtype. Moreover, both genes (combined or separately) show biomarker potential to discriminate BL and LumA from non-tumoral and BL from LumA, supporting *NORAD* as the most relevant lncRNA with PUM binding sites in BC and the molecular axis where *NORAD*, PUM and RALGAPB participate as a potential target for novel BC targeting strategies [41]. 

Neuropilin 1 (NRP-1) transcript and protein levels were associated with BC progression, with increased levels in BC cell lines [64], higher expression in TNBC compared to LumB [65], poorer BC prognosis [66] and higher treatment resistance [67]. In BC, *miR-376a* was reported to have decreased expression in circulation [68], tumors [69] and several cell lines, and it is positively associated with OS. Indeed, *miR-376a* OE suppressed BC cell proliferation, migration and invasion and increased apoptosis, through direct binding to *NRP-1* [70]. *PUM2* knockdown (KD) in MDA-MB-231 and MDA-MB-453 cell lines showed the attenuation of stemness properties, such as decreased expression of aldehyde dehydrogenase 1 (ALDH1) family member A1 and Nanog homeobox (NANOG) proteins, lower ALDH1 activity and decreased spheroid formation capacity. Bioinformatic analysis and luciferase assays revealed that both PUM2 and *miR-376a* bind to the 3′UTR region of *NRP-1*. Mechanistically, PUM2 and *miR-376a* compete for *NRP-1* binding, with PUM2 promoting BC stemness and *miR-376a* attenuating it. PUM2 can then induce the expression of NRP-1 by binding its mRNA and thus regulate BC progression [46].

Differential alternative polyadenylation (APA) was previously reported to be altered in BC tumors [71], and increased expression of polyadenylation components, like cleavage stimulation factor subunit 3 (*CSTF3*), was detected in TNBC cell lines. Several mRNAs with different prevalence of 3′ UTR isoforms, such as shortened and lengthened 3′UTR regions, were detected in BC tumors. It was found that PRE is the most frequently lost motif in shortened 3′UTRs in BC, but also the most often gained through APA. This suggests that PRE-containing RNAs are frequently altered by APA. Moreover, BL and TNBC tumors present more extensive and exclusive patterns of APA than LumA and LumB tumors. Gene Ontology (GO) analysis of the APA-exclusive alterations in TNBC tumors showed that the transcripts are related to the negative regulation of apoptosis, kinase activity and nucleotide binding. For instance, forkhead box O1 (*FOXO1*), a tumor suppressor transcription factor from the FOXO family group, showed extended 3′ UTR, whereas the tumor suppressor phosphatase and tensin homolog (*PTEN*), the proto-oncogene Neuroblastoma RAS viral oncogene homolog (*NRAS*) and the Jun proto-oncogene (*c-JUN*) showed recurrent 3′ UTR shortening, the latter two being the most recurring alterations. Overall, this study suggests that the dysregulated expression of *PTEN*, *NRAS*, *c-JUN* and *FOXO1* in BC relies on increased or decreased PRE-bound PUM-regulation [47], with PUM playing an important part in regulating relevant cancer-related signaling pathways.

*MiR-323a-3p* is a miRNA related to tumor resistance, with decreased expression in BC tissues and cell lines and tumor suppressor roles in neuroblastoma [72] and esophageal squamous cell carcinoma (ESCC) [73]. The downregulation of *miR-323a-3p* in BC cell lines results in increased viability, migration and invasion and the opposite upon *miR-323a-3p* OE. Bioinformatics and experimental assays such as RNA pulldown uncovered *NORAD* and *miR-323a-3p* binding. Moreover, *NORAD* expression directly influences *miR-323a-3p* levels, and a decrease in *miR-323a-3p* expression promotes *NORAD*-induced aggressive behavior in MDA-MB-453 cells. Bioinformatic database (Targetscan, DIANA and Starbase) analysis and RNA pulldown assays revealed that PUM1, which displays increased levels in BC tumors and cell lines, binds to *miR-323a-3p*. Indeed, *NORAD* OE impacts PUM1 expression, and *PUM1* depletion reverses the proliferation, migration and invasion capacities induced by upregulated *NORAD*, while *miR-323a-3p* negatively regulates PUM1 levels [30]. In this study, it was shown that both *NORAD* and *miR-323a-3p* can influence PUM1 and eukaryotic translation initiation factor 2 alpha kinase 3 (PERK)/eukaryotic initiating factor 2 (eIF2)/activating transcription factor 4 (ATF4) PERK/eIF2/ATF4 signaling pathway as *NORAD* OE decreases p-PERK, p-eIF2 and ATF4 protein levels. In vivo xenograft mouse models established with *NORAD*-depleted or *miR-323a-3p*-overexpressing BC cell lines reveal reduced size and weight of xenograft tumors and increased apoptosis as measured by TUNEL assay. Immunohistochemistry analysis of xenografts’ tumor sections confirmed that in vivo *NORAD* inhibition results in increased *miR-323a-3p* and p-PERK and decreased PUM1 levels. In sum, *NORAD* inhibition or *miR-323a-3p* OE can decrease BC cell malignant behavior by inhibiting PUM1 and activating the downstream eIF2 signaling pathway [30]. 

A study using transcriptomics analysis from invasive breast carcinoma surgical tissue samples revealed the downregulation of *NORAD* in BL when compared to the LumA subtype. Survival analysis did not render any significant differences, but higher levels of *NORAD* were associated with lower DFS only in BL patients. Despite that, *NORAD* promoted accessibility, as measured using ATAC-seq, whereas methylation, from genome-wide methylation studies, was not significantly altered between the BL and LumA subtypes. Transcriptomic analysis from TCGA highlights *NORAD* as the central regulator for regulon reconstruction, revealing a network of co-expression with genes potentially modulated by *NORAD*, some of them being PUM target genes, such as the proteasome assembly chaperone 4 (*PSMG4*) [45], a proteasome assembly chaperone protein upregulated in lung neoplastic cells and correlated with poor prognosis [74]. *NORAD* regulon showed a positive activity in ER+ and PR+ tumors but was inactive in BL tumor samples. Moreover, molecular signatures and GO analysis did not reveal any significant terms between the networks of BL and LumA tumor samples, but the pathways observed were closely linked to luminal epithelial cell transformation, including BMP and ALK1 signaling. *NORAD* is thus differently expressed in BC subtypes and participates in a complex regulatory network alongside many PUM target genes [42].

Secretory carrier membrane protein 1 (*SCAMP1*) is a lncRNA that promotes cancer progression through cell viability and invasion [75]. The *SCAMP1* variant 2 (*SCAMP1-TV2*) shows increased expression in BC tumors from both LumA and TNBC subtypes and in several human BC cell lines, where *SCAMP1-TV2* silencing promotes decreased levels of PI3K and AKT, both phosphorylated and unphosphorylated forms. Evidence suggests that *SCAMP1-TV2* binds PUM2, which in turn targets INSM transcriptional repressor 1 (*INSM1*), which is able to inhibit SAM and SH3 domain containing 1 (*SASH1*), which can finally influence PI3K/AKT signaling [48]. INSM1 is a protein that regulates MYC proto-oncogene (c-Myc) and promotes BC carcinogenesis [76]. INSM1 expression is increased in human BC, and it has been proposed as a prognostic neuroendocrine marker for LumB [77,78,79]. In this study, *INSM1* OE promoted increased MDA-MB-231 and MCF-7 BC cell viability, migration and invasion and decreased apoptosis. Moreover, it reversed the BC inhibitory effects of *PUM2* OE and was accompanied by decreased expression of SASH1, a protein with tumor suppressor activity in TNBC involved in the toll-like receptor 4 (*TLR4*) signaling pathway [80,81,82,83]. Additionally, *SASH1* OE decreased BC cell viability, migration and invasion and PI3K and AKT levels, while it increased apoptosis. In vivo tumor xenograft mice models established by the inoculation of MCF-7 or MDA-MB-231 cell lines with several combinations of *SCAMP1-TV2* and PUM2 expression revealed that the simultaneous silencing of *SCAMP1-TV2* and PUM2 OE renders the highest inhibition of xenograft tumor growth [48]. PUM2 proves, yet again, its importance and broad range of targets and its ability to influence cancer-related signaling pathways.

### 2.2. NORAD-Regulated Signaling Pathways via ncRNA Sponging

There are various classes of ncRNAs, namely, transfer RNAs (tRNAs), ribosomal RNAs (rRNAs), small RNAs (sRNAs) and lncRNAs [6]. ncRNAs can create complex networks by interacting with each other, affecting cancer cell fate and survival through different mechanisms, being considered promising diagnostic, prognostic biomarkers and therapeutic targets in cancer [84]. In particular, lncRNAs are the most predominant and diverse class among all ncRNAs [6]. They can interact with different biological molecules, such as DNA, RNA, including other ncRNAs, and proteins [84]. On the other hand, miRNAs can regulate gene expression by cleaving RNA or repressing the translation of their mRNA targets, thus regulating several biological processes such as cell cycle progression, proliferation, apoptosis and development [6]. LncRNAs can, however, act as ceRNAs by binding to miRNAs and suppress their targeting of mRNAs [85]. Next, we will describe examples of ncRNAs regulated by *NORAD* with an impact on BC progression (summarized in Figure 1 and Table 1). The impact of *miR-323a-3p*, a *NORAD*-binding miRNA, was previously discussed in the context of PUM target genes (see Section 2.1).

The upregulation of *miR-155-5p* has been associated with the malignant behavior of BC cells. *MiR-155-5p* is implicated in BC by targeting suppressor of cytokine signaling 1 (*SOCS1*), a key regulator of cell proliferation and apoptosis that plays a crucial role in the degradation of ubiquitination substrates. Notably, SOCS1 acts as a tumor suppressor by facilitating the degradation of oncoproteins, inhibiting cell proliferation and apoptosis [86]. The reduced expression of *SOCS1* is linked to poor prognosis in BC patients, leading to lower OS rate as compared to high-*SOCS1*-expression patients. In the human HCC70 BC cell line, *NORAD* seems to work as a tumor suppressor through its capability to sponge *miR155-5p*, which leads to the positive regulation of *SOCS1* and a reduction in cell proliferation, migration and invasion behavior in vitro, affecting overall BC progression [38].

*MiR-590-3p* has been described as a tumor suppressor in several cancers [87,88,89]. In BC cells, *miR-590-3p* OE is associated with the inhibition of proliferation and higher apoptosis [87]. Moreover, *miR-590-3p* inhibits Golgi phosphoprotein 3 (GOLPH3), a protein associated with a poor prognosis and chemoresistance in BC patients [90], suggesting that *miR-590-3p* can regulate BC progression through the regulation of GOLPH3. Mechanistically, the lncRNA *NORAD* can function as a sponge to *miR-590-3p*, negatively regulating its expression and oncogenic function in the context of BC. The depletion of *NORAD* or *miR-590-3p* OE resulted in decreased MCF-7 and MDA-MB-231 BC cell proliferation, invasion and migration in vitro, with a concomitant decrease in GOLPH3 protein levels, indicating that *NORAD* might be involved in BC pathophysiology by mediating the *miR-590-3p*/GOLPH3 signaling axis [32].

A study analyzing the differently expressed transcripts between normal and TNBC, HER2+, LumA and LumB tumors predicted that *NORAD* could promote the occurrence and development of BC tumors. It proposes that *NORAD* accomplishes this by interacting with other ncRNAs like metastasis-associated lung adenocarcinoma transcript 1 (*MALAT*1) and sponging several miRNAs, including *miR-183*, *miR-182*, *miR-7*, *miR-149*, *miR-200c*, *miR-101* and *miR-342*. In turn, these miRNAs can regulate the expression of key signaling pathways, as forkhead box O3 (*FOXO3*) and ras homolog family member A (*RHOA*) [50]. The reduced expression of both *FOXO3* and *RHOA* is associated with clinical outcomes in BC, namely, metastasis, BC cell proliferation and tumorigenesis [91,92,93]. In this context, *NORAD* levels also correlate with *RHOA* and RAD51 antisense RNA 1 (*RAD51-AS1*) expression. *NORAD* is significantly increased in BC tumors compared to adjacent normal tissue, presenting a great specificity value for segregation between BC and non-tumoral tissues [51].

### 2.3. Protein- and mRNA-Mediated Regulation of Signaling Pathways by NORAD

The transforming growth factor β (TGF-β), mitogen-activated protein kinase (MAPK) and the response to DNA damage are major signaling pathways in BC. *NORAD* was shown to regulate these pathways through the differential interaction with numerous mRNA and protein partners. In particular, the *MAPK14*, a member of the MAPK family, has been described to promote BC tumor progression [94,95,96]. Although there was no significant difference in either *NORAD* or *MAPK14* levels between tumors and adjacent normal tissue, *NORAD* was shown to be significantly correlated with *MAPK14* expression in BC tumors [49]. Hereafter, we will describe other *NORAD* interactions that can play crucial roles in BC (Figure 1 and Table 1).

TGF-β is a highly conserved family whose signaling is involved in different cellular processes such as cell growth, proliferation, migration and differentiation [97,98]. TGF-β signaling can either suppress or induce tumor progression, as it promotes cell cycle arrest and apoptosis in early BC stages, whereas in advanced stages, it favors cell motility, invasion and epithelial-to-mesenchymal transition (EMT) [99]. A study by Zhou et al. revealed that the upregulated expression of *NORAD* in human BC cells and patient tumors is associated with increased cell proliferation, migration and invasion in vitro and worse patient survival, by influencing the TGF-β signaling pathway. Silencing *NORAD* expression in BC cell lines leads to decreased TGF-β protein expression and the downregulation of its downstream effectors, such as SMAD family member 2 (Smad2) and RUNX family transcription factor 2 (RUNX2). In this way, *NORAD* promotes BC progression by regulating the TGF-β signaling pathway [35], highlighting the potential control of *NORAD* as a key tumor-suppressive event in BC.

In the context of BC therapy, the treatment of the TNBC MDA-MB-231 human cell line with doxorubicin triggers sustained DNA damage signals via H2A.X variant histone (H2AX) phosphorylation. Double-strand break amplification culminates in the recruitment of DNA damage signaling and repair proteins, such as BRCA1 DNA repair-associated protein (BRCA1) and tumor protein TP53 binding protein 1 (53BP1), to the damaged sites [52,99,100]. In the absence of *NORAD*, cells persist in signaling DNA damage via H2AX phosphorylation which may stem from an aberration either downstream or upstream of *NORAD*. Upon *NORAD* depletion, MDA-MB-231 cells show decreased levels of PARP1, impairing the DNA damage repair [52]. Noteworthy, PARP inhibitors are currently employed in treating advanced-stage metastatic BC particularly in cases with germline mutations in *BRCA1* or *BRCA2* genes, frequently associated with the TNBC subtype [100].

The yes-associated protein (YAP)/WW domain containing the transcription regulator 1 (TAZ)–TEA domain transcription factor (TEAD) complex is shown to be inversely correlated with *NORAD* expression in breast-invasive carcinoma in TCGA [39]. TEAD3 and TEAD4 are the anchor proteins of this complex, which are modulated by the Hippo signaling pathway, controlling cell growth and cancer progression [101]. TEAD4 was found to bind the *NORAD* promoter in the 5′ regulatory region of *NORAD* and silencing of *TEAD1/3/4* resulted in increased *NORAD* expression in the human TNBC Hs578T cell line [39]. YAP, TAZ and the NuRD-repressive complex [102] and other components, including metastasis-associated protein (*MTA1)* and chromodomain helicase DNA binding protein 4 (*CHD4*), were all recruited to that same region of *NORAD* promoter. Furthermore, silencing *MTA1* and *CHD4* led to further *NORAD* upregulation, confirming that YAP/TAZ and NuRD repress *NORAD* transcription. On the other hand, *NORAD* repression by the YAP/TAZ pathway contributes to the YAP/TAZ-mediated promotion of migration and invasion in the BC-mutated cell line Hs578 YAP 8SA [39], where YAP is inactive and cannot be phosphorylated [100,103]. *NORAD* silencing in the human ZR75 luminal BC cell line increased S100P association with the IQ motif containing GTPase activating protein 1 (IQGAP1) and TP53 proteins, while *NORAD* OE attenuated this interaction. In the human TNBC MDA-MB-231 cell line, the specific binding of S100P protein and *NORAD* was observed, with S100P OE reversing *NORAD* OE and S100P silencing counteracting *NORAD* depletion. A similar relationship was observed in vivo, where MDA-MB-231 *NORAD*-overexpressing cells, upon tail vein i.p. injection, formed fewer lung metastatic nodules compared to control or *NORAD*/S100P double KD cells. In this context, although *NORAD* is shown to be transcriptionally repressed by YAP/TAZ-TEAD, *NORAD* also sponges S100P to inhibit metastasis [39].

### 2.4. NORAD-Regulated Cytokines and Immune Cells

The tumor microenvironment (TME) plays a major role in BC progression and therapy response [104]. In particular, CD8 T immune cells are crucial in anticancer immune response [105], where a higher amount of CD8 T-infiltrating lymphocytes (TILs) predicts a better immunotherapy response [106] and high levels of CD8 T-cells in samples correlate with better BC prognosis [53]. *NORAD* expression in BC tissues is also proven to be correlated with the TME, immune infiltration and expression of immune checkpoint inhibitors [31]. The impact of *NORAD* in immune cell regulation during BC progression and in the therapy response will be highlighted below.

A study using data from TCGA, which divided BC samples into high and low CD8 T-cell numbers, revealed that *NORAD* expression was elevated in the low CD8 T-cell group and high-risk BC samples, with smaller OS rate. Moreover, *NORAD* was negatively correlated with the presence of CD8 T-cells, cytotoxic lymphocytes and T-cells in the tumor, while it was positively associated with the levels of fibroblasts, endothelial cells and neutrophils. *NORAD* expression was also negatively related to immune checkpoint genes such as lymphocyte-activating 3 (*LAG3*), T-cell immunoreceptor with Ig and ITIM domains (*TIGIT*), cytotoxic T-lymphocyte-associated protein 4 (*CTLA4*) and programmed cell death 1 (*PDCD1*) [53]. *NORAD* co-expresses with several targets of immune regulation signaling pathways such as cytokines and interleukins (ILs), as TGF-β, *IL-3*, *IL-4* and Type I Interferon [36]. These data show a connection between *NORAD* expression and immune cell regulation in BC, including CD8 T-cell numbers, which can potentially be modulated to improve therapy response.

In BC, *NORAD* expression was found to be preferentially related to macrophage regulation, which shows a preferential upregulation of M2-polarized protumoral CD206-expressing macrophages, in comparison with M1-polarized antitumoral CD68-expressing macrophages. A study revealed that macrophage polarization can be directed by TNBC cell line-derived exosome internalization. In comparison to macrophages incubated with exosomes derived from normal breast epithelium MCF-10A cells and *NORAD*-depleted MDA-MB-231 cells, MDA-MB-231-derived exosome co-culture with non-polarized macrophages resulted in higher levels of *NORAD* and expression of M2 markers (*CD163*; mannose receptor C type 2, *MRC2*; Arginase 1, *Arg1*) and lower expression of M1 markers (*CD80*; C-C motif chemokine ligand 2, *MCP-1*; nitric oxide synthase 2 *iNOS*). Moreover, macrophages previously incubated with *NORAD*-depleted MDA-MB-231-derived exosomes, when co-cultured with MDA-MB-231 cells, promoted several effects in the BC cells, including decreased expression of *NORAD*, reduced proliferation, migration and invasion and increased apoptosis. Moreover, silencing *NORAD* in macrophages decreased the expression of TGFB1 and phosphorylated Smad2 and 3, potentially through *miR-92b-3p*, that binds both *NORAD* and TGFB1. These results show that *NORAD* can contribute to the activation of macrophages that promote malignant behavior in BC cells [36].

## 3. Potential Implication of *NORAD* in BC Therapies

As previously discussed, elevated *NORAD* levels have mostly been associated with BC aggressiveness and poor RFS in patients. Conversely, *NORAD* KD has shown inhibitory effects on BC cell viability and migration in vitro [30] and in vivo cancer progression [35]. Considering the association between *NORAD* and genomic instability, together with the paradoxical role of CIN in tumor progression, Alves-Vale et al. explored the possibility of simultaneously targeting *NORAD* together with cytotoxic drugs in BC treatment. Proteome analysis by liquid chromatography–tandem mass spectrometry (LC/MS-MS) revealed that *NORAD* KD in TNBC cells produced a significant alteration in the modulation of proteins associated with DNA repair, chromatin remodeling and epigenetic regulation. This suggests a potential impact on sensitivity to DNA-damaged agents such as doxorubicin. Of note, a significant decrease in the levels of minichromosome maintenance complex component 6 (MCM6), a critical player in DNA replication initiation, and Aly/REF export factor (ALYREF), a known interactor of *NORAD* associated with poor survival in BC patients, indicate a potential influence on cancer cell survival and therapy response [43]. Moreover, combinatorial *NORAD*/*PARP1* silencing in the presence of doxorubicin in MDA-MB-231 cells had a synergistic effect on the abnormal accumulation of phosphorylated H2AX (γH2AX), a marker of DNA damage. These observations suggest that *NORAD* might confer BC resistance to chemotherapeutic agents, further impacting sensitivity to treatment [43]. Similar to *NORAD*, the lncRNA *H19* has been implicated in BC chemoresistance, and *H19* upregulation in doxorubicin-resistant BC cells correlates with decreased sensitivity to chemotherapy. Silencing *H19* expression has been shown to sensitize doxorubicin-resistant MCF-7 cells to chemotherapy, indicating a potential similar function of *NORAD* and highlighting lncRNA targeting in sensitizing BC therapy-resistant cells [107].

*NORAD* has the potential to significantly enhance the effectiveness of chemotherapy, presenting an opportunity to improve treatment outcomes [43]. Radiotherapy is often used in BC patients following surgery and systemic chemotherapy [108]. It exerts its action by inducing mainly DNA double-stranded breaks (DSBs), promoting cell apoptosis and inhibiting cell cycle progression [109]. Thus, the DNA damage machinery has a key role in resistance to radiotherapy. DNA damage can be repaired by homologous repair (HR) and non-homologous end joining (NHEJ), and alterations in these pathways can lead to radiotherapy-resistant tumors [110]. In ESCC, *NORAD* depletion can be used in combination with radiotherapy for sensitizing radiotherapy-resistant ESCC cells in a colony formation assay, suggesting that *NORAD* can be an effective target to enhance the cytotoxic effects of radiation in ESCC patients [111]. Efforts in the development of combined approaches using immunotherapy in cancer treatment have been rising in recent years. Sun et al. 2021 conducted a study where the inhibition of DNA repair machinery was used to increase the immune checkpoint therapy efficiency combined with radiotherapy in ESCC cells. Using an in vivo xenograft model with *NORAD*-depleted KYSE-150 cells, the authors showed that increased *NORAD* expression was correlated with ESCC cells’ radio-resistance and that a combination of radiation with antiprogrammed death-1 (PD-1) antibodies was able to decrease tumor growth in *NORAD*-depleted tumor-bearing mice. These efforts highlight the potential of targeting *NORAD* in combination with radiotherapy in promoting a more efficient response to immunotherapy in cancer treatment [112]. Although no studies combining *NORAD* depletion with radiotherapy and immunotherapy in BC patients have been conducted, these data highlight the potential use of *NORAD* in sensitizing BC patients to combined therapies, such as radiotherapy [111]. Based on the reported studies, we envision that several currently available therapies could potentially benefit from *NORAD* depletion (summarized in Table 2). The impairment of the DNA damage response machinery is a key point in all the strategies, with a more direct impact on therapies using PARP inhibitors. When targeting the FOXO1 pathway, *NORAD* KD has the potential to synergize with the expected molecular outcomes of the therapy. *NORAD* KD is also associated with increased sensitivity to several therapies, which opens the possibility of improving tumor response to treatments with, for instance, PI3K/AKT/mTOR (PAM) inhibitors in combination with doxorubicin.

## 4. Conclusions and Future Perspectives

The lncRNA *NORAD* has been associated with the progression of several cancers. Compelling evidence suggests that *NORAD* is implicated in a myriad of signaling pathways relevant for BC, such as TGF-β, PI3K/AKT and FOXO1. Its dysregulation has been observed in a spectrum of cancers, particularly in BC, where its OE is mostly associated with increased proliferation, invasion, metastasis, resistance to chemotherapy, poor patient prognosis and OS. By sequestering PUM1 and PUM2, *NORAD* indirectly regulates the expression of PUM targets. In this line of thought, all the described effects of PUM in BC can be indirectly regulated by *NORAD*, increasing its therapeutic potential. Due to its predominant oncogenic role, it would be especially relevant to test the potential of targeting *NORAD* in a neoadjuvant therapy setting to sensitize and potentiate BC treatment. 

Although some studies discriminated among BC subtypes, the majority only analyzed bulk BC samples, many with small sample sizes. As lncRNAs present a tissue-specific expression and *NORAD* has been shown to act differently according to the BC subtype, it would be important to further discern the role of *NORAD* in the different BC subtypes more accurately. In addition, the majority of conducted studies of *NORAD* and PUM targets in BC were performed using 2D cell line models, disregarding 3D cell–cell and cell–matrix interactions of cancer, stroma and immune cells in the tumor microenvironment, of outmost importance for cancer progression. Thus, it would be important to deepen our knowledge of *NORAD* in BC in more complex models that better mimic the native BC tumor.

The synergy observed between *NORAD* depletion and chemotherapeutic agents suggests a promising path for enhancing BC treatment outcomes (Table 2). The involvement of *NORAD* in radio-resistance, as observed in ESCC, opens possibilities for its exploitation in combination with radiotherapy for BC treatment. Several immune checkpoint inhibitors, such as CTLA4, PD-1 and PD-L1 have received approval from the FDA for treating solid tumors, including BC. The correlation between the number of TILs and favorable prognoses in HER2+ and TNBC is significant, with a potential to decrease the risk of relapse and death to 15–25% [117]. The impact of *NORAD* on immune regulation and correlation with immune checkpoint markers and its ability to influence the response to immunotherapy highlight its potential role in enhancing the efficacy of immunotherapy in BC. We suggest that *NORAD* depletion may improve immunotherapy results through different mechanisms. By disrupting its role in maintaining genomic stability, *NORAD* KD may induce increased genomic instability, leading to the generation of more neoantigens that enhance tumor immunogenicity [118]. This heightened immunogenicity could render the tumor more susceptible to immunotherapeutic interventions, particularly those targeting neoantigens recognized by the immune system. Furthermore, *NORAD* KD may enhance immunogenic cell death, a process triggered by DNA damage, potentially increasing the release of danger signals that attract immune cells to the tumor site [119], thereby improving the effectiveness of immunotherapies. Moreover, reduced *NORAD* levels may sensitize cancer cells to immunotherapeutic interventions, particularly immune checkpoint inhibitors, by modifying cellular processes that contribute to immune evasion [120].

The future of *NORAD* in BC therapies holds promise, emphasizing the need for continued research to unravel its intricate mechanisms. As we navigate the complexities of BC treatment, *NORAD* emerges as a potential biomarker by distinguishing BC subtypes to better assist clinical decision, being a potential neoadjuvant therapeutic target, as its silencing allows for the sensitization of BC cells to chemotherapy, and being a key player in shaping the landscape of personalized and targeted interventions against BC. As *NORAD* interacts with RNAs, coding and non-coding and proteins but most of the studies are performed exploring PUM in BC, it would be interesting to further explore the vast array and relevance of *NORAD* in other signaling pathways relevant to BC progression. The ongoing exploration of the role of *NORAD* in BC will open new possibilities for improved patient outcomes and a deeper understanding of the molecular intricacies concerning BC progression and treatment.

## Figures and Tables

**Table 1:** Target genes, proteins and pathways affected by NORAD expression in breast cancer. N/A stands for non-applicable.

Targets	In Vitro	In Vivo	Mechanism of ACTION	BC Impact	References
PUM/PSMG4	N/A	Human bioinformatic database (TCGA) and tumors	*NORAD* targets PUM, PUM targets PSMG4, and *NORAD* co-expresses with PSMG4	Lower DFS in BL	[45]
PUM2/ *miR-376a*/ NRP-1	Human cell lines (MCF-7, T47D, MDA-MB-231, MDA-MB-453, HMEpC)	Human tumors	*NORAD* targets PUM; PUM2 and *miR-376a* competitive-bind to NRP-1	Higher cell stemness	[46]
PUM/ RALGAPB	N/A	Human bioinformatic databases (TCGA, cBioPortal)	*NORAD* co-expresses with RALGAPB and PUM targets RALGAPB	Worse prognosis and poor OS in LumA; subtype biomarker	[41]
PUM/ c-JUN, FOXO1, NRAS, PTEN	Human cell lines (SK-BR-3, MDA-MB-231, CAL51, BT-20, BT-549)	Human bioinformatic database (GEO) and tumors	*NORAD* targets PUM; PUM targets c-JUN, FOXO1, NRAS and PTEN	Lower cell proliferation and invasion	[47]
PUM1/ eIF2/ PERK/ ATF4	Human cell lines (MCF10A, MCF-7, MDA-MB-231, MDA-MB-468, MDA-MB-453, T47D)	Human tumors and cancer xenograft mouse models	*NORAD* targets PUM; PUM1 targets eIF2/PERK/ATF4	Suppression of tumor growth; lower cell viability, proliferation, migration and invasion	[30]
S100P	Human cell lines (293FT, MDA-MB-231, Hs578T, T47D, ZR75)	Human bioinformatic databases (TCGA, GEO, PROGgeneV2) and tumors, cancer mouse models	*NORAD* binds S100P, preventing its binding to *TP53* and *IQGAP1*	Suppression of migration, invasion and metastasis	[39]
PUM2/ INSM1/ SASH1/ PI3K/AKT	Human cell lines (MCF-10A, MCF-7, MDA-MB-231)	Human tumors and cancer xenograft mouse models	*NORAD* targets PUM; PUM2 targets INSM1, decreasing SASH1 repression and inhibiting PI3K/AKT	Lower cell viability, migration, invasion and tumor growth and reduced apoptosis	[48]
MAPK14	N/A	Human bioinformatic databases (HGNC, lncBase v2, Expression Atlas, Co-lncRNA) and tumors	*NORAD* co-expresses with MAPK14	Biomarker	[49]
*miR-155-5p* and SOCS1	Human cell lines (HCC70, MCF-7, SKBR-3 and T-47D)	N/A	*NORAD* targets *miR-155-5p*, preventing its binding to SOCS1	Lower cell proliferation and invasion	[38]
*miR-590-3p* and GOLPH3	Human cell lines (MCF-7, MDA-MB-231, T47D, BT-549)	Human tumors	*NORAD* targets *miR-590-3p*, preventing the degradation of GOLPH3	Higher cell proliferation, invasion and migration and lower apoptosis	[32]
miRNAs/FOXO3 and RHOA	N/A	Human bioinformatic database (GEO)	*NORAD* interacts with *miR-183*, *miR-182*, *miR-7*, *miR-149*, *miR-200c*, *miR-101* and *miR-342*, regulates FOXO3 and RHOA	Biomarker	[50,51]
γ-H2AX	Human cell lines (MCF-7, MDA-MB-231, MDA-MB-436, MDA-MB-468)	N/A	*NORAD* recruits DDR proteins that repair damage through the phosphorylation of H2AX	Lower cell proliferation and invasion	[52]
TGF-β/ RUNX2	Human cell lines (MDA-MB-231 and MCF-7)	Human tumors and cancer mouse models	*NORAD* depletion decreases TGF-β protein expression	Higher cell proliferation, invasion and migration and worse prognosis	[35]
Immune cells	Human cell lines (MCF-10A, MDA-MB-231)	Human bioinformatic database (TCGA) and tumors	*NORAD* higher in low CD8 T-cell count; the promotion of malignant M2 macrophage polarization by exosome internalization	Poorer prognosis, higher tumor progression	[36,53]

**Table 2:** Examples of BC therapies that might benefit from NORAD depletion.

Therapy	BC Application	Mechanism of Action	Barriers to Therapy Response	Potential Impact of *NORAD* Depletion	Impact of *NORAD* Depletion in Therapy Response	References
PARP inhibitors	BRCA mutations	Impairment of SSB repair	Restoration of HR	Improved PARP downregulation and impairment of DDR	Inhibition of tumor cell growth and proliferation	[52]
DNA damage-inducing chemotherapy	First-line therapy	DNA damage leads to apoptosis and inhibition of proliferation	DNA damage repair and resistance to therapy	Potential synergistic effect on FOXO1 downregulation	Reinforcement of apoptosis and inhibition of proliferation	[113]
FOXO1 inhibitor (AS1842856)	BL tumors	FOXO1 pathway inhibition	Inhibitor does not bind to the phosphorylated form of FOXO1	Potential synergistic effect on downregulating FOXO1 and its phosphorylated form	Reinforcement of apoptosis and inhibition of proliferation	[114]
PAM inhibitors combined with CDK4/6 inhibitors	ER+ tumors	PAM downregulation leads to the diminished capability of BC to acquire resistance to endocrine therapy	Acquired resistance to endocrine therapy	mTOR inhibition	Improved sensitization of tumor cells to endocrine therapy	[115]
PAM inhibitors combined with anti-HER2 antibodies	HER2+ tumors	PAM downregulation sensitizes to anti-HER2 antibodies	Acquired resistance to HER2 antibodies	Synergistic effect on downregulating PAM	Improved sensitization of tumor cells to HER2 antibodies	[115]
Doxorubicin	First-line therapy	DNA DSB and activation of RhoA/MLC pathway	Promotes migration and invasion via RhoA/MLC pathway	Impairment of DNA damage repair machinery	Decreased tumor cell survival and inhibition of migration and invasion	[116]

DDR—DNA damage repair; DSB—double-strand break; HR—homologous repair; SSB—single-strand break.
